# Psychological features of adult patients with langerhans cell histiocytosis

**DOI:** 10.1371/journal.pone.0246604

**Published:** 2021-02-12

**Authors:** Emmanuelle Bugnet, Nishant Gupta, Gwenaël Lorillon, Sayena Arbabzadeh-Bouchez, Cédric Lemogne, Sylvie Chevret, Abdellatif Tazi

**Affiliations:** 1 Assistance Publique-Hôpitaux de Paris, Hôpital Saint-Louis, Centre National de Référence des Histiocytoses, Service de Pneumologie, Paris, France; 2 Division of Pulmonary, Critical Care and Sleep Medicine, University of Cincinnati, Cincinnati, Ohio, United States of America; 3 Department of Veterans Affairs, Veterans Affairs Medical Center, Cincinnati, Ohio, United States of America; 4 Assistance Publique-Hôpitaux de Paris, Hôpital Fernand Widal, Service de Psychiatrie, Paris, France; 5 Université de Paris, F-75006, Paris, France; 6 Assistance Publique-Hôpitaux de Paris, Hôpital Européen Georges Pompidou, Service de Psychiatrie de l’Adulte et du Sujet âgé, Paris, France; 7 Assistance Publique-Hôpitaux de Paris, Hôpital Saint-Louis, Service de Biostatistique et Information Médicale, Paris, France; 8 INSERM UMR-1153 (CRESS), Biostatistics and Clinical Epidemiology research team (ECSTRRA), Paris, France; Mansoura University, EGYPT

## Abstract

**Background:**

The prevalence of psychological symptoms and the co-occurrence of substance abuse disorders in adult patients with Langerhans cell histiocytosis (LCH) has not been previously explored. We aimed to use validated scales to evaluate depression and anxiety symptoms experienced by adult LCH patients.

**Methods:**

In this cross-sectional study, all consecutive adult LCH patients seen at our national reference center between January 2012 and January 2013 were asked to complete the following instruments: the Hospital Anxiety and Depression scale (HADS); Barratt Impulsiveness Scale, Version 10 (BIS-10); and Cannabis Use Disorders Identification Test (CUDIT). Self-reported scores on these scales were used to determine the point prevalence of clinically significant psychological symptoms and substance use disorders in LCH patients. Patient profiles in terms of psychological features were assessed by principal component analysis including the HADS and BIS-10 instruments values, followed by hierarchical clustering. Fisher exact tests and Wilcoxon tests were used to examine the associations between disease-related parameters and high levels of anxiety and impulsivity.

**Results:**

Seventy-one adult LCH patients, mainly with pulmonary LCH (PLCH), completed the evaluations. Clinically significant anxiety and depression symptoms were reported by 22 (31%) and 4 (6%) subjects, respectively. Impulsivity was detected in 14% (10/71) of the patients. Seventeen percent (12/71) of the patients used cannabis on a regular basis, with 50% of these individuals (6/12) exhibiting scores consistent with cannabis use disorder. Three derived clusters of patients were identified in the principal component analysis; these patient clusters differed in successful weaning from tobacco at the time of evaluation (p = 0.03). In univariate analyses, isolated PLCH and the use of psychotropic treatments were statistically associated with clinically significant anxiety symptoms.

**Conclusions:**

High levels of anxiety and impulsivity are common in adult patients with LCH. The consequences of these symptoms for the management of LCH patients warrant further evaluation.

## Introduction

Langerhans cell histiocytosis (LCH) is an inflammatory myeloid neoplasm driven by the abnormal accumulation of specialized dendritic cells carrying pathogenic activating mutations in the mitogen activating protein (MAP) kinase pathway [1–3]. LCH can be a systemic disorder affecting multiple organ systems and typically develops in childhood [2]. Pulmonary LCH (PLCH) can be seen either in association with systemic disease or, more commonly, as an isolated entity typically encountered in young to middle-aged adults [4]. The prevalence of PLCH is unknown. Early studies with a confirmed histologic diagnosis highlighted the rarity of the disease, which accounted for 3 to 5% of adult patients with diffuse infiltrative lung diseases [5,6]. However, these reports most probably underestimated the true prevalence of PLCH, as with the wide use of chest high resolution computed tomography (HRCT) in the evaluation of patients, lung biopsy is not performed in all cases of PLCH [4]. The most striking epidemiologic feature of PLCH is that it occurs almost exclusively in smokers or ex-smokers (>90% of cases) [4]. No other epidemiologic factors associated with PLCH have been identified. The role of smoking in triggering PLCH is highlighted by the finding that most children with systemic LCH who developed PLCH in adolescence or adulthood started to smoke before this event [7]. Recently, a murine model elegantly showed that exposure of mice to cigarette smoke was necessary to induce lung lesions similar to those observed in PLCH [8].

Based on the strong association with cigarette smoke exposure, smoking cessation has been the mainstay of management in patients with PLCH [4,9,10]. However, achieving successful and durable cessation of exposure to cigarette smoke has historically been very difficult in patients with PLCH, and the natural history of this population is characterized by multiple relapsing and remitting episodes with regards to cigarette smoking [11]. The clinical course of PLCH is highly unpredictable, and a significant proportion of patients continue to deteriorate even after successful smoking cessation, leading to permanent disabling consequences such as respiratory failure, pulmonary hypertension (PH), and the need for lung transplantation [4,12,13]. As is true for any patients with chronic disabling conditions, patients with PLCH may experience significant emotional distress resulting from the psychological impact of the diagnosis that is exacerbated by the rarity of the disease and the resultant lack of knowledge about the disease and the related impairments. In this regard, a significant impairment of health quality of life has been reported in LCH patients, although the underlying psychological features, such as anxiety and depression, were not assessed in these studies [14–17].

In addition to addiction to nicotine, PLCH patients have also been shown to engage in excessive alcohol [18] and cannabis consumption [4,19]. Comorbid substance use is likely to further increase the distress associated with the disease [20]. Reciprocally, it has been shown that people with depression or anxiety disorders are less likely to successfully quit smoking [21,22]. Although, the interplay among depression, anxiety and smoking is likely to affect the clinical course of the disease, these aspects in adult patients with LCH, including those with PLCH, have not been systematically evaluated.

As a first step to unravel this issue, we conducted an exploratory study with the aim of using validated scales to examine the depression and anxiety symptoms experienced by adult LCH patients seen at the French National Reference Center for Histiocytoses. In addition, since high levels of impulsivity have been linked to several addictive behaviors, including chronic tobacco smoking and cannabis use [23], this dimension was also measured in the present study.

## Materials and methods

### Study design and subject selection

In this cross-sectional study, all consecutive adult patients with LCH seen at the National Reference Center for Histiocytoses between January 2012 and January 2013 were eligible for the study, provided they could complete the self-reported instruments. Clinical data of the study patients were prospectively registered in the database of the French Registry for Histiocytoses and retrospectively analyzed.

The diagnosis of LCH was either histologically confirmed by a biopsy of an involved site or, for patients with PLCH, was based on a typical lung high-resolution computed tomography (HRCT) pattern, either alone or in association with characteristic extrapulmonary involvement (i.e., lytic bone lesion, diabetes insipidus) and the exclusion of alternative diagnoses [13]. The study was performed in accordance with the principles of the amended Helsinki Declaration and approved by the Institutional Review Board of the French Institute of Medical Research and Health (IRB number 00003888). All patients provided written informed consent for the use of their medical reports for research.

### Instruments

Patients completed the following self-reported instruments: the Hospital Anxiety and Depression Scale (HADS); Barratt Impulsiveness Scale, Version 10 (BIS-10); and Cannabis Use Disorders Identification Test (CUDIT).

The HADS is a 14-item instrument. This scale is a brief and reliable assessment tool for the detection and evaluation of the overall severity of anxiety- and depression-related symptoms during the past week. Seven questions each related to anxiety and depression are rated on four-point scales (0–3). Each anxiety or depression subscale ranges from 0 to 21; a score ≥ 11 denotes clinically significant depression or anxiety symptoms [24].

The Barratt Impulsiveness Scale, French Version 10 (BIS-10), is a 34-item instrument that evaluates 3 main impulsiveness domains: motor (defined as acting without thinking), cognitive (making fast decisions), and nonplanning (lack of future planning). All questions are rated on a four-point scale (rarely/never, occasionally, often, and almost always/always). Answers are scored as 0, 1, 3, and 4, where 4 indicates the most impulsive response. Thus, the total BIS-10 score may vary from 0 to 136, with a median score of 68 [25,26].

The CUDIT is a 10-item instrument that measures the patient’s cannabis use over the past six months. Items 1 to 8 are rated on a five-point scale (0–4), and items 9 and 10 are scored as either 0 or 4. The maximum attainable value on the CUDIT instrument is 40. Cannabis use disorder is indicated by a score of 8 or more [27].

### Research procedures

Data on patient demographics, smoking habits, cannabis and alcohol consumption, psychotropic medication(s) use, clinical symptoms and signs, lung function and LCH organ localizations at the time of study inclusion were retrieved from the database. Stratification of LCH was performed according to the Histiocyte Society criteria to differentiate between single-system (SS) and multisystem (MS) disease [28]. LCH treatments received by the patients prior to the time of inclusion in the study were also recorded.

At the time of evaluation, patients were classified as having nonactive disease in the case of the resolution of all symptoms and signs. Otherwise, they were considered to have active disease, which was further categorized into regressive (improvement of symptoms or signs, with no new lesions), stable (persistence of symptoms or signs, with no new lesions) or progressive (progression and/or appearance of new lesions) disease. For patients with symptomatic lung involvement, disease state classification was based on the pulmonary function test (PFT) trends observed during the 12-month time frame prior to study inclusion [11]. In patients for whom prior PFTs were not available, symptomatic progression based on the New York Heart Association (NYHA) stage of dyspnea and/or the occurrence of a spontaneous pneumothorax were taken into consideration when classifying patients into the regressive, stable, or progressive categories [11]. Patients who were on long-term supplemental oxygen and/or had developed respiratory failure and/or PH were classified as having progressive lung disease. Patients who developed new extrapulmonary disease manifestations, such as new endocrine dysfunction, were classified as having progressive disease. The presence of associated disabling comorbidities (such as malignancy, symptomatic cardiovascular disease, chronic obstructive pulmonary disease (COPD), etc.) were recorded. Finally, the management plan proposed to the patients at the time of the study was also recorded.

The primary endpoint of the study was the proportion of patients having clinically significant levels of anxiety, as defined by a HADS-A score of 11 or higher. Secondary outcomes were the scores on the HADS-D, BIS-10, and CUDIT tests. A threshold value of 68, which corresponds to the median total score on the BIS-10 scale, was used to define high levels of impulsivity.

### Statistical analysis

Summary statistics that included the mean and standard deviation or median with interquartile range [IQR] or percentages were calculated.

First, principal component analysis (PCA) was used to identify psychological patterns based on the different instrument values (HADS-A, HADS-D, HADS-total, BIS-10 subscores and total). Briefly, PCA is an unsupervised exploratory multivariate learning method that gives a global representation of the scores without any prior knowledge about whether the samples came from different groups or had phenotypic differences [29]; it is usually regarded as statistically significant when the cumulative value of the first three components is above 60%. Underlying assumptions, that is multinormality and linear relationships between all variables were checked using Mardia’s test [30] and scatterplot, respectively. Sample adequacy was measured by the Kaiser-Meyer-Olkin factor [31]. Last, McDonald’s omega was used to measure reliability of the principal components [32]. Then, based on the principal components, ascending hierarchical clustering (AHC) was carried out to identify clusters. The number of clusters was chosen based on the natural break in distance jumps. To characterize the clusters, descriptive statistics for the score values and outcomes were computed.

Second, comparison tests, namely Fisher exact tests for binary and qualitative variables, and Wilcoxon tests for quantitative variables, were used to assess the existence of associations of various disease-related parameters and anxiety, depression or impulsivity profiles, as defined above.

Statistical analyses were performed using SAS (SAS Inc, Cary, NC, USA) and R (https://www.R-project.org/) software. All tests were two-sided, with p-values < 0.05 denoting statistical significance, with no adjustment for multiple comparisons.

## Results

### Characteristics of the patients

No patient declined to participate in the study. Seventy-one patients, with a median age of 44.3 years [IQR, 33.5–49.0] were included in the study; 41 (58%) were females. The diagnosis of LCH was histologically confirmed in 39 patients. The remaining patients had typical lung HRCT patterns (associated with diabetes insipidus or bone involvement for 4 and 2 patients, respectively). The median time between LCH diagnosis and inclusion in the study was 48.1 months [IQR, 10.6–102].

The characteristics of the patients at the time of the study are detailed in Table 1. Forty-five patients were active smokers at the time of study inclusion (all with lung involvement), and 26 patients were nonsmokers (among whom 19 had successfully weaned themselves from using tobacco since receiving the diagnosis). Twelve patients had used cannabis during the past six months. Fifty patients had SS LCH (1 with bone involvement and 49 with lung involvement), and 19 had MS disease (all with lung involvement) and two patients with isolated bone involvement at the time of diagnosis did not have active LCH at the time of study inclusion. Thirteen (18%) patients had received systemic LCH treatment (median time 40 months [IQR, 17–103]) before study participation.

**Table 1:** Characteristics of the LCH patients at the time of inclusion in the study.

Baseline characteristic	*n*	*%*	*median*	*IQR*
Age in years	71	100	44.3	33.5–49.0
Female	41	58		
Time from diagnosis, months			48.1	10.6–102
Current smokers	45	63		
+ cannabis consumption	12	17		
Weaned from tobacco since diagnosis	19	27		
Regular consumption of alcohol ^a^	9	14		
Psychotropic treatment ^b^	13	18		
SS LCH	50			
Lung	49			
Bone	1			
MS LCH ^c^	19			
Lung +				
DI	13			
Alone	9			
with AHD	4			
Bone	10			
Liver	3			
Skin	2			
Peripheral node	1			
Resolved disease	2			
Symptoms				
None	30	42		
Dyspnea	35	49		
NYHA II/III	25/10			
Bone pain	5	7		
Disabling skin lesion	2	3		
Lung function	66			
Restriction ^d^	6	9		
Obstruction	15	23		
Air trapping	29	44		
D_LCO_ <80% (n = 65)	60	92		
LCH systemic treatment since diagnosis	13	18		
Vinblastine + steroids	4			
Steroids	3			
Cladribine	5			
alone	1			
after steroids	2			
after vinblastine + steroids	2			
Biphosphonate	1			

^a^ Information was available for 66 patients, defined as ≥40 g/day consumption of alcohol. One of these patients also consumed cannabis.

^b^ Antidepressant (alone n = 7, with anxiolytic n = 2); anxiolytic alone (n = 4).

^c^ Some patients had more than 2 organs involved.

^d^ Restriction was defined as a TLC<80% of predicted, obstruction as FEV1/FVC<70% and air trapping as RV/TLC>120% of predicted.

*Abbreviations*: AHD: Anterior hypophysis hormonal deficiency, DI: Diabetes insipidus, D_LCO_: Diffusion capacity of carbon monoxide, FEV_1_: Forced expiratory volume in one second, FVC: Forced vital capacity, LCH: Langerhans cell histiocytosis, MS: Multisystem, NYHA: New York Heart Association, PH: Pulmonary hypertension, RV: Residual volume, SS: Single system, TLC: Total lung capacity.

The disease state at the time of inclusion is shown in Table 2. Among the 68 patients with lung involvement, 14 had progressed during the previous year, including 7 with respiratory failure and/or PH. Disabling comorbidities were present in 11 patients (Table 2).

**Table 2:** Disease state at the time of inclusion in the study of the 71 LCH patients.

Baseline characteristic	*n*	*%*
Overall disease state	71	100
NAD	2	3
AD regressive	17	24
AD stable	25	49
AD progressive	17	24
Lung involvement outcome (n = 68)	68	96
Improved	16	23
Stable	38	56
Worse ^a^	14	21
Comorbidities ^b^	11	15
Lymphoma	1	
CMML	1	
Thyroid cancer	1	
Benign cerebral tumor	1	
Cardiovascular disease	3	
COPD	2	
Obesity	1	
Narcolepsy	1	
Ulcerative colitis	1	
Cluster headache	1	
Future management plan	71	100
Observation	63	89
Cladribine	3	4
Lung transplantation	5	7

^a^ Seven of these patients had respiratory failure and/or PH.

^b^ 2 comorbidities were present in 2 patients.

*Abbreviations*: Same as Table 1 plus AD: Active disease, CMML: Chronic myelomonocytic leukemia, COPD: Chronic obstructive pulmonary disease, NAD: Nonactive disease.

At the time of evaluation, the future management plan consisted of observation for 63 (89%) patients (with pulmonary rehabilitation for 2 patients), cladribine was proposed to 3 patients, and lung transplantation was considered for 5 patients with chronic hypoxemic respiratory failure.

### Self-reported instrument scores

The results of the 3 instruments are shown in Table 3. Seventy-one patients completed the HADS and the BIS-10, and the 12 cannabis consumers completed the CUDIT.

**Table 3:** Scores on HADS, BIS-10 and CUDIT scales in the study population.

Baseline characteristic	*n*	*%*	*median*	*IQR*
**HADS** ^a^	71	100		
**Median anxiety score**			8	6–12
Clinically significant anxiety symptoms	22	31		
Score			13	12–15.5
** Median depression score**			4	2–6
Clinically significant depression symptoms	4	6		
Score			11.5	11–12.75
**BIS-10** ^b^	71	100		
Median total score			46	38–56.5
Motor			13	9–20
Cognitive			16	13–20
Nonplanning			16	11–21
Total score >68	10	14		
**CUDIT** ^c^	12	17		
**Median score**			7.5	2–13.75
Cannabis use disorder	6	50		
Score			15.5	11.25–20.5

^a^ Values range from 0 to 21 with values ≥ 11 denoting clinically significant anxiety or depression symptoms.

^b^ Values range from 0 to 136 (higher score denotes higher level of impulsiveness); 68 is the median value of the scale.

^c^ Values range from 0 to 40, with values ≥ 8 indicating cannabis use disorder.

*Abbreviations*: HADS: Hospital Anxiety Depression Scale, BIS -10: Barratt impulsiveness scale, IQR: Interquartile range, CUDIT: Cannabis Use Disorders Identification Test.

According to the HADS, the median anxiety score was 8 [IQR, 6–12], and 22 (31%) patients had clinically significant anxiety symptoms. The median depression score was 4 [IQR, 2–6], and clinically significant depression symptoms were identified in 4 (6%) patients.

The cumulative median BIS-10 score of the whole study population was 46 [IQR, 38–56.5]. Ten (14%) patients had a score greater than 68, i.e., greater than the median value for the scale. The subscale scores for the motor, cognitive, and nonplanning domains are detailed in Table 3.

The median CUDIT score of the 12 patients who consumed cannabis was 7.5 [IQR, 2–13.75]. Six out of the 12 cannabis-consuming patients (50%) had a score ≥ 8 (median score 15.5 [IQR 11.25–20.5]), suggesting cannabis use disorder (Table 3).

Comparison of the instrument values according to the smoking status of the patients at inclusion, showed no significant association with tobacco use (HADS: p = 0.16; BIS-10: p = 0.06; CUDIT: p = 1.00).

### Principal component analysis

**Fig 1:**
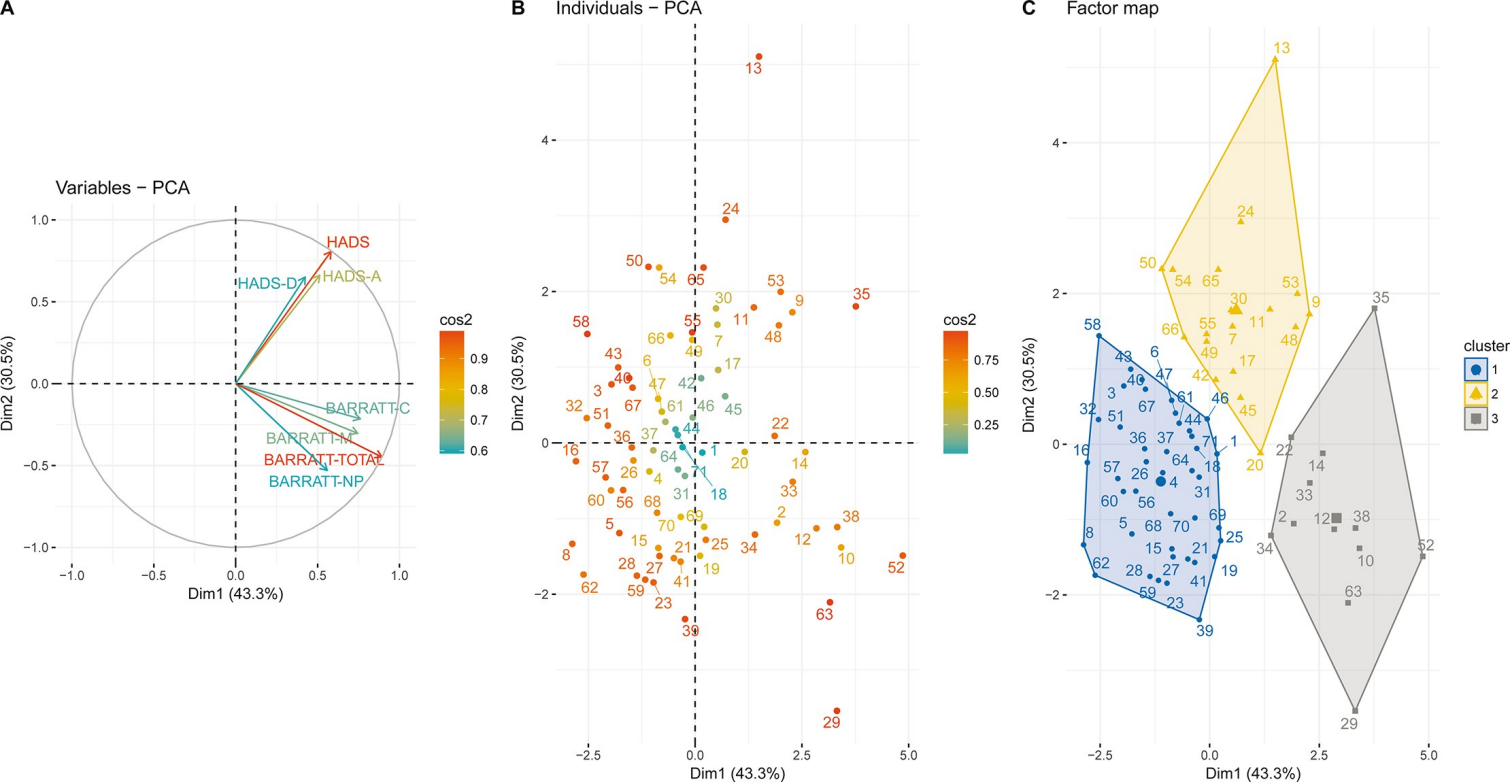
Principal Component Analysis (PCA) of the HADS and BIS-10 scores of 71 LCH patients. Loading plots of the first two components are shown: the first component is on the horizontal axis, and the second component is on the vertical axis. (A) Plots of the HADS and BIS-10 variables. (B) Plots of patients. (C) Clusters identified among the study population through hierarchical clustering analysis.

After checking for multinormality (with Mardia’s skewness of 4.9 and kurtosis of -1.19, in agreement with such a distribution), as well as linear relationships and absence of outliers as illustrated on scatterplots, we performed the PCA using the 3 instruments. The two first components of the PCA accounted for more than 70% of the total variance. Fig 1 shows the representation of subjects with regard to the first two components of the PCA (scores plot). The first axis (43.3% contribution) seems to reflect high values for both the HADS and the BIS-10 scores, while on the second axis (12.6% contribution), high HADS scores were associated with low BIS-10 scores. Values of McDonald’s omega was found to be 0.97 for the total dimensions of the PCA. However, as expected given the small sample size, the Kaiser-Meyer-Olkin factor adequacy was of 0.42, suggesting exploratory rather than definite findings.

Following PCA, three clusters were identified that appeared to be reflective of (i) patients free from any symptoms of anxiety, depression and impulsivity (cluster 1, median HADS and BIS-10 scores of 10 and 42, respectively), (ii) patients with high levels of anxiety symptoms (cluster 2, median HADS of 19.5 vs. BIS-10 of 44.5), and (iii) patients with high levels of impulsivity (cluster 3, median BIS-10 score of 74 vs. HADS of 15). The three clusters also differed in terms of successful weaning from tobacco, with quit rates of 41%, 24% and 0% in clusters 1, 2 and 3, respectively (p = 0.03).

### Factors associated with self-reported instrument scores

Table 4 shows the results of univariable analyses of the factors associated with clinically significant anxiety symptoms in the study population at the time of evaluation. Isolated PLCH and the use of psychotropic treatment were statistically associated with clinically significant anxiety symptoms.

**Table 4:** Univariate analyses of the factors associated with clinically significant anxiety symptoms in the study population at the time of evaluation.

	*No anxiety (n = 49)*				*Anxiety (n = 22)*				*p*
Characteristic	*n*	*%*	*median*	*IQR*	*n*	*%*	*median*	*IQR*	
Age			44	31–48			46	37–50	0.32
Gender									
Female	25	51			16	73			0.12
Male	24	49			6	27			
Current tobacco smokers	29	66			16	80			0.58
Weaned from tobacco since diagnosis ^a^	15	34			4	20			
Time from diagnosis			53	10–120			41	12–64	0.47
LCH classification									
SS ^b^	31	63			19	86			0.049
MS	17	35			2	9			
Resolved disease	1	2			1	5			
Symptoms									
Yes	28	57			12	55			
No	21	43			10	45			1.00
Dyspnea									
Yes	24	49			11	50			1.00
No	25	51			11	50			
Lung function ^c^									
Obstruction	12	26			3	15			0.52
Restriction	4	9			2	10			1.00
Air trapping	19	41			10	50			0.59
Overall disease state									
AD regressive	9	18			8	36			0.11
AD stable	24	49			11	50			
AD progressive	15	31			2	9			
NAD	1	2			1	5			
Lung involvement outcome ^d^									
Improved	9	19			7	35			0.24
Stable	27	56			11	55			
Worse	12	25			2	10			
Comorbidities									
Yes	8	16			3	14			1.00
No	41	84			19	86			
Future plan management									
Observation	42	86			21	95			0.69
Cladribine	3	6			0	0			
Lung transplantation	4	8			1	5			
Psychotropic treatment	2	4			11	50			< .0001

Note. Clinically significant anxiety defined by an HADS-A score of 11 or higher.

^a^ At the time of diagnosis, 64 patients were current smokers.

^b^ Isolated PLCH was present in 49 of the 50 LCH patients with SS disease.

^c^ TLC, RV/TLC and FEV_1_/FVC were available for 46 and 20 patients, in the nonanxiety and anxiety groups, respectively.

^d^ Lung involvement was present in 68 of the 71 patients studied (48 and 20 in the nonanxiety and anxiety groups, respectively). Longitudinal pulmonary function tests were available in 50 patients.

*Abbreviations*: Same as Table 1.

Table 5 shows the results of univariable analyses of factors associated with the BIS-10 scores. No predictive factor was associated with the BIS-10 score, considering a threshold value greater than 68 as indicative of impulsivity, based on the median value.

**Table 5:** Univariate analyses of the factors associated with barratt scores, considering a threshold score value greater than 68, corresponding to the median total score on the scale.

	*Low levels of impulsivity (n = 61)*				*High levels of impulsivity (n = 10)*				*p*
Characteristic	*n*	*%*	*median*	*IQR*	*n*	*%*	*median*	*IQR*	
Age			43.9	35–48.7			47.3	30.4–49.7	0.84
Gender									
Female	34	56			7				0.50
Male	27	44			3				
Current tobacco smokers	37	66			8	100			0.09
Weaned from tobacco since diagnosis	19	34			0				
Time from diagnosis			52.3	11.8–108.4			39.6	3.8–57.4	0.25
LCH classification									
SS ^a^	45	74			5	50			0.12
Resolved disease	1	2			1	10			
MS	15	24			4	40			
Symptoms									
Yes	34	56			7	70			0.50
No	27	44			3	30			
Dyspnea									
Yes	28	46			7	70			0.19
No	33	54			3	30			
Lung function ^b^									
Obstruction	14	25			1	11			0.67
Restriction	4	7			2	22			0.18
Air trapping	26	46			3	33			0.72
Overall disease state									
AD regressive	15	25			2	20			0.20
AD stable	32	52			3	30			
AD progressive	13	21			4	40			
NAD	1	2			1	10			
Lung involvement outcome ^c^									
Improved	14	24			2	22			0.56
Stable	34	58			4	45			
Worse	11	18			3	33			
Comorbidities									
Yes	10	16			1	10			1.00
No	51	84			9	90			
Future plan management									
Observation	54	88			9	90			0.72
Cladribine	3	5			0	0			
Lung transplantation	4	7			1	10			
Psychotropic treatment	10	16			3	30			0.38

Note. At the time of diagnosis, 64 patients were current smokers.

^a^ Isolated PLCH was present in 49 of the 50 LCH patients with SS disease.

^b^ TLC, RV/TLC and FEV_1_/FVC were available for 57 and 9 patients, in the low and high levels of impulsivity groups, respectively.

^c^ Sixty-eight patients had lung involvement (59 and 9 in the groups with low and high levels of impulsivity, respectively). Longitudinal pulmonary function tests were available in 50 patients. Restriction was defined as a TLC<80% of predicted, obstruction as FEV_1_/FVC<70% and air trapping as RV/TCL>120% of predicted.

*Abbreviations*: Same as Table 1.

## Discussion

The main results of our study are as follows: 1) anxiety symptoms are common in adult patients with LCH, with an estimated prevalence of 31%; 2) high levels of impulsivity were present in 14% of LCH patients; and 3) cannabis use disorder is common in patients with LCH, as it was identified in 17% of our study population at the time of evaluation.

This is the first study evaluating the presence of anxiety and depression symptoms, levels of impulsivity and prevalence of cannabis use disorder in adult patients with LCH, as confirmed by the search in PubMed using relevant keywords. Indeed, although impairment of health quality of life and cognitive alterations have been reported in LCH patients, particularly in children, evaluation of anxiety and depression has not been previously specifically assessed in these patients [14–17]. Interestingly however, a significant proportion of patients with Erdheim-Chester disease, a non-Langerhans histiocytosis affecting predominantly adults, was reported to complain from anxiety and depression, based on a dedicated scale developed for this rare disease [33].

The HADS was originally developed by Zigmond and Snaith in 1983 to detect reported anxiety and depression symptoms in patients evaluated at nonpsychiatric clinics. It was specifically designed to exclude the influence of other somatic symptoms that might be present due to the underlying physical ailments rather than reflecting psychological problems associated with anxiety and depression [24]. This scale has subsequently been validated in multiple studies spanning a spectrum of populations ranging from the general population to populations with various underlying health conditions, including those in psychiatric clinics, with a sensitivity and specificity for identifying anxious and depressive states ranging between 78–90% [34]. There is a strong link between cigarette smoking and mental disorders such as anxiety and depression, with some authors suggesting that nicotine dependence may be a means of self-medication for patients with anxiety and depression seeking to control their psychological symptoms [35]. However, this hypothesis may only account for substance use initiation, as tobacco smoking has been shown to subsequently increase the risk of both depression and anxiety disorders [20,22]. In our study, clinically significant levels of anxiety were more common in patients with SS LCH, i.e., most commonly PLCH, as opposed to MS LCH. The high prevalences of PLCH and nicotine dependence in our cohort suggest that this association may be driven largely by nicotine addiction, as suggested by our PCA. However, we did not identify any significant association between smoking status and clinically significant anxiety symptoms in our study population in the univariable analysis.

The BIS scale, originally developed in 1995 in the United States [25], is one of the most widely used scales to measure impulsive personality traits and has been adapted for use in multiple countries across the world, including the French translation utilized in our study [26]. The measurement of impulsivity is relevant to this particular population, as impulsive behavior traits have been linked to a variety of substance use disorders, including nicotine [23]. Interestingly, no patient who had been successfully weaned from tobacco at the time of evaluation in the present series had high levels of impulsivity. Notably, there is an inverse relationship between the presence of impulsivity and retention in treatment programs for substance use disorders [36]. Assessing specific areas of impulsivity and targeting interventions aimed at altering these traits might assist in improving the overall quit rates for cigarette smoking in patients with PLCH.

The CUDIT questionnaire was developed by modifying the Alcohol Use Disorders Identification Test and initially employed to screen for the presence of concomitant cannabis use in patients with mild to moderate alcohol dependence [27]. As such, the validity of this measure has mainly been established in at-risk populations rather than in the general population [27]. Given the high prevalence of cannabis abuse in patients with PLCH [4,19], we propose that the PLCH population is reflective of a high-risk group in which the application of the CUDIT questionnaire would be considered a valid extrapolation.

High levels of anxiety and depression are frequently seen in patients with chronic diseases and have a substantial negative impact on overall health [37]. For instance, the prevalences of anxiety and depression in patients with chronic respiratory disorders such as COPD have been estimated to be approximately 36% and 40%, respectively [38]. Similar estimates of anxiety and depression approaching a prevalence of 30% have been described in patients with other chronic diseases, such as type I diabetes mellitus [39] and sarcoidosis [40]. While the overall prevalence of anxiety symptoms was similar in our cohort of patients with PLCH, the prevalence of depression symptoms appeared to be lower than in patients with other chronic disorders. The exact reasons for this discrepancy are unknown but might relate to the relatively small sample size of our population and differences in the mode of ascertainment (type of scale used). When compared to the general population, the prevalence of anxiety symptoms was higher in our LCH patients, whereas the prevalence of clinically significant depression symptoms in these patients was comparable to the prevalence of major depression in the general population [41].

Smoking cessation remains the cornerstone of management in patients with PLCH [4]. Not only does successful smoking cessation promote physical disease stabilization and/or regression [11,42,43] but also the results of our nonsupervised PCA suggest associations between tobacco smoking and anxiety and impulsivity symptoms in patients with PLCH. However, achieving successful abstinence from cigarette smoke exposure has proven to be quite challenging, with a rate of approximately 20% achieved after 24 months of prospective follow-up in a prospective multicenter study of PLCH patients [11]. Overall, these rates are similar to the long-term abstinence rates observed in other randomized clinical trials evaluating pharmacological approaches to smoking cessation, including nicotine replacement therapy, bupropion, and nicotine receptor partial agonists such as varenicline [44,45]. Integrating other nonpharmacological approaches such as behavioral support along with pharmacological treatment may offer an additive benefit with regard to achieving successful smoking cessation [46] and needs to be further investigated in patients with PLCH, along with other novel approaches that can leverage the current technology such as mobile phones and the internet [47,48].

The role of nicotine as a gateway drug that can lead to the initiation and persistence of addiction to other substances has been well established [49]. The coexistence of alcohol and cannabis abuse in a substantial proportion of our cohort (14% and 17%, respectively) is likely a manifestation of this phenomenon and further highlights the critical importance of early, aggressive measures aimed at curbing nicotine addiction in these patients to avoid future substance use disorders.

This study has several limitations. This was a single-center study, which raises the question of its external validity. Further studies are needed before extrapolating our results to LCH patients in general. The relatively small sample size of our cohort also limits the statistical power, and our analysis may have failed to detect additional differences among the patients. Moreover, we did not perform any multivariable analysis given the small number of patients with clinically significant anxiety and impulsivity, and thus confounding factors cannot be excluded. Last, PCA requires several underlying assumptions to be fulfilled. While multinormality of the scores and linear relationship between all of them were not ruled out, the sample size of the study population was not large enough to provide reliable results. Therefore, our results should be considered exploratory.

To the best of our knowledge, this is the first study aimed at evaluating anxiety and depression symptoms, impulsivity and substance use disorder in adult LCH patients. The major strengths of this study include the use of validated instruments and a detailed clinical assessment of the disease state at a national reference center for histiocytic disorders. These results provide the framework for further investigation of neuropsychological involvement in LCH and will promote the integration of these and other validated screening methods in future natural history cohorts and the clinical evaluation of these patients. Replication and validation of these assessments in geographically diverse regions of the world is needed to further advance this field.

## Conclusions

Clinically significant symptoms of anxiety and high levels of impulsivity are common in adult patients with LCH. The impact of these disturbances on the natural history of the disease course and the management of LCH, especially with regards to addictive behavior, warrants further investigation.
